# Editorial: Drugs and methods that enhance the anti-cancer efficacy of artesunate

**DOI:** 10.3389/fphar.2025.1566700

**Published:** 2025-03-17

**Authors:** Tuğçenur Uzun

**Affiliations:** Samsun Oral and Dental Health Hospital, Samsun, Türkiye

**Keywords:** artesunate, artemisinin, cancer, autophagy, ferroptosis, reactive oxygen species (ROS), PI3K/AKT/mTOR, non-coding RNA

“Ginghao, one bunch, take two sheng of water for soaking it, wring it out, take the juice, ingest it in its entirety.” This prescription, found in A Handbook of Prescriptions for Emergencies written by Ge Hong in AD 400, was recommended for treating “intermittent fever,” a symptom of malaria (Hsu, 2006). Centuries later, this prescription laid the foundation for the discovery of artemisinin, a groundbreaking molecule in malaria treatment. Artemisinin and its derivatives have saved millions of lives, marking a turning point in medical history.

Recent scientific studies suggest that the therapeutic potential of artemisinin and its derivatives has not yet been fully explored. Beyond their role in malaria treatment, these compounds may serve as important, effective, and safe agents in cancer therapy. Artesunate, the sodium salt of the hemisuccinate ester of artemisinin, is synthesized to improve its pharmacokinetic properties. The addition of the hemisuccinate group enhances its water solubility, allowing for both intravenous and oral administration, while also facilitating rapid absorption and systemic distribution, thereby reinforcing its potential in cancer treatment (Presser et al., 2017; Simpson et al., 2006). The anticancer effects of artesunate were first demonstrated by Dr. Efferth et al. in 2007, and by Krishna et al. in 2008. These effects were subsequently validated in numerous *in vivo* and *in vitro* experiments (Krishna et al., 2008; Efferth et al., 2007). Thus, this story, which began with an ancient prescription, continues to open new horizons in the medical field.

Artesunate exerts its anticancer effects by influencing various cellular mechanisms. For instance, it has been shown to induce apoptosis and autophagy in human bladder cancer cells (Zhou et al., 2020; Zhao et al., 2020). Additionally, it has been reported to cause cell cycle arrest, reactive oxygen species (ROS) generation, and ferroptosis in renal cell carcinoma (Markowitsch et al., 2020).

Ferroptosis, a regulated cell death mechanism triggered by iron-dependent lipid peroxidation, has emerged as a promising target in cancer therapy. This process is distinct from other forms of cell death, such as apoptosis, necrosis, and autolysis, due to its unique morphological and biochemical characteristics. The high iron demand and susceptibility of cancer cells to ferroptosis make this mechanism particularly advantageous in addressing drug resistance and metastasis. Liu et al. have explored the role of ferroptosis in therapy resistance and metastasis, detailing the molecular mechanisms associated with ferroptosis and its potential in overcoming therapy resistance and preventing metastasis (Liu et al.).

Several studies have shown that reduced sensitivity to ferroptosis leads to therapy resistance in cancer cells (Hangauer et al., 2017; Gao et al., 2016). Conversely, inducing ferroptosis has been found to enhance the efficacy of standard chemotherapy, targeted therapies, radiotherapy, and immunotherapy (Wen et al., 2024; Jiang et al., 2020). Furthermore, biological processes such as epithelial-mesenchymal transition (EMT) and non-coding RNAs have been shown to regulate ferroptosis, thereby influencing metastasis (Luo et al., 2021; Zuo et al., 2022; Zhang et al., 2024). In this context, targeting ferroptosis represents a crucial strategy for both preventing cancer spread and re-sensitizing resistant cells to therapy.

As an antimalarial drug, artesunate has recently gained attention from cancer researchers for its effects on ferroptosis (Song et al., 2022; Li Z. J. et al., 2021). Artesunate triggers ferroptosis by increasing ROS production and disrupting iron metabolism (Li Z. J. et al., 2021; Pang et al., 2016). Particularly in ferroptosis-sensitive cancer types, the cytotoxic effects of artesunate are further enhanced when combined with standard treatments such as platinum-based drugs, paclitaxel, and temozolomide (Li W. et al., 2021; Tran et al., 2017; Karpel-Massler et al., 2014).

The potential of artesunate in cancer therapy extends beyond overcoming drug resistance. It also has the capacity to inhibit cancer spread by inducing ferroptosis in metastatic cells. For example, by suppressing EMT and increasing ROS production, artesunate offers a significant advantage in targeting metastatic cancer cells.

While ferroptosis-based mechanisms offer promising strategies in cancer treatment, the development of therapeutic approaches requires innovative designs to diversify and enhance efficacy. In this regard, combining different pharmacophores provides a novel pathway to address fundamental challenges such as drug resistance and selective toxicity. Pharmacophore hybrids represent tools that not only complement existing treatment strategies but also expand the clinical benefits of current mechanisms by targeting multiple pathways while sparing healthy tissues.

The combination of distinct active pharmacophores offers significant advantages, including modulation of multiple targets, enhanced efficacy, prevention of resistance development, and reduction of side effects. Dong et al. designed and synthesized 15 hybrids by combining dihydroartemisinin and isatin derivatives with a three-carbon linker, demonstrating their potent anti-lung cancer activity. These hybrids were extensively tested for efficacy against drug-sensitive (A549), doxorubicin-resistant (A549/DOX), and cisplatin-resistant (A549/DDP) lung cancer cell lines (Dong et al.).

Biological evaluations revealed that hybrids 6a and 6e exhibited comparable IC50 values to doxorubicin and cisplatin in both drug-sensitive and resistant lung cancer cell lines. Furthermore, their non-toxic profile in normal lung epithelial cell lines highlighted their selectivity. Notably, hybrid 6a not only displayed high efficacy against cancer cells but also demonstrated good stability in mouse and human microsomes and superior pharmacokinetic properties, making it a promising candidate for preclinical studies.

The study by Dong et al. underscores the innovative potential of developing pharmacophore hybrids in cancer therapy. These hybrids offer novel solutions for enhancing therapeutic efficacy while overcoming challenges such as drug resistance and toxicity. They are anticipated to hold a significant place in future clinical applications.

In their study, Lin and Chen discussed the diverse biological activities and therapeutic potential of artemisitin (ATT), a natural compound isolated from Artemisia annua L. and an analogue of artemisinin. ATT, identified as an endoperoxide, has shown promise not only in antimalarial effects but also in diseases such as inflammation, oxidative stress, and cancer. For instance, ATT has been shown to bind to cysteine residues of the Keap1 protein, stabilize Nrf2, and reduce oxidative stress-induced lung damage. Additionally, ATT has been reported to inhibit NLRP3 inflammasome activation, thereby suppressing inflammatory processes. In the context of anticancer effects, ATT has been shown to induce ferroptosis and target NEDD4/c-Myc/topoisomerase pathways, thereby suppressing tumor growth. However, the unclear target proteins of ATT remain a significant barrier to its clinical application. Lin and Chen emphasize that identifying ATT’s target proteins is critical for structural optimization and elucidation of its pharmacological mechanisms.

Ma et al. comprehensively investigated the anticancer effects of artesunate (ART) on choroidal melanoma (CM) using network pharmacology, molecular docking, and experimental validation. The study addressed the limited treatment options and poor prognosis associated with CM, a highly malignant ocular tumor.

Artesunate, has exhibited broad-spectrum antitumor properties. The researchers identified potential ART targets using network pharmacology and highlighted key mechanisms such as apoptosis induction, cell cycle arrest, and oxidative stress regulation. Molecular docking studies confirmed ART’s strong binding ability to proteins associated with these pathways.

Experimental validation supported the anticancer effects of ART. *In vitro* experiments demonstrated that ART induced apoptosis via the p53 signaling pathway, inhibited the PI3K/AKT/mTOR pathway to arrest the cell cycle at the G0/G1 phase, and increased ROS levels to activate the NRF2/HO-1 signaling pathway. These findings were corroborated by *in vivo* studies, which showed significant suppression of CM tumor growth by ART.

The study also explored ART’s potential to inhibit tumor metastasis. ART was proposed to interfere with tumor migration through interactions with MMP2 and MMP9, which play critical roles in extracellular matrix degradation and metastasis.

In conclusion, Ma et al. presented compelling evidence of ART’s multifaceted anticancer effects on CM by targeting oncogenic pathways, promoting apoptosis, arresting the cell cycle, and increasing ROS levels. These findings highlight ART as a promising agent for CM treatment, paving the way for future therapeutic strategies.

The pharmacological scope of artemisinin and its derivatives extends beyond malaria treatment, offering promising opportunities for managing complex diseases like cancer. The multifaceted mechanisms of these molecules create significant potential for their integration into current therapeutic strategies. This scientific journey, bridging traditional knowledge and modern biotechnological innovation, demonstrates the profound outcomes that can arise from such a synthesis. Successfully translating these therapeutic strategies into clinical applications requires addressing key challenges, including the optimization of drug delivery systems, elucidation of resistance mechanisms, and improvement of formulation strategies to enhance bioavailability and therapeutic efficacy. Moreover, a deeper understanding of molecular targets and the clarification of safety profiles are essential to ensure their effective and safe clinical implementation. In this context, artemisinin derivatives are expected to play a pivotal role in developing more effective and targeted therapies.
